# Supplementary material for: Removal of Methylene Blue from Water Using Magnetic GTL-Derived Biosolids: Study of Adsorption Isotherms and Kinetic Models
Source: Molecules. 2023 Feb 3;28(3):1511. doi: 10.3390/molecules28031511 (PMC9919137; doi:10.3390/molecules28031511)
Supplement: Supplementary file 1 [file molecules-28-01511-s001.zip › molecules-2101603-supplementary.pdf]

Title: Removal of methylene blue from water using magnetic GTL-derived biosolids: Study of adsorption isotherms and kinetic models

Table S1. *Activated carbon properties*

Sample	Surface area (m^2/g)	Pore volume (cm^3/g)
Activated BS (1:1 impregnation ratio)	25.12 ± 1.5	0.09 ± 0.01
Activated BS (1:2 impregnation ratio)	47.23 ± 2.1	0.12± 0.01

Table S2. *Studied isotherm models*

Isotherm models			
**Model name**	**Equation**	**Description**	**Reference**
Langmuir	$q_e = \frac{K_L C_e}{1 + a_L C_e}$	Considers adsorption as a continuous bombardment of molecules onto a surface with their corresponding desorption or evaporation from the surface with no aggregation at the surface	[1]
Freundlich	$q_e = a_F C_e^{b_F}$	Not limited to monolayer formation and can be applied to formation of multilayers. Adsorption heat does not need to be uniformly distributed on the heterogeneous surface of the isotherm	[2]
Redlich-Peterson	$q_e = \frac{K_R C_e}{1 + a_R C_e^{b_R}}$	Can be applied to heterogenous and homogenous systems as it features both Freundlich and Langmuir models	[3]
Langmuir-Freundlich (LF/ SIPS)	$q_e = \frac{K_{LF} C_e^{n_{LF}}}{1 + a_{LF} C_e^{n_{LF}}}$	Combines Langmuir and Freundlich isotherm models to predict the heterogeneity of the system- it localizes the adsorption without adsorbate–adsorbate interaction	[4]
Toth	$Q_e = \frac{Q_m C_e}{[K_T + C_e{}^n)]^{1/n}}$	Is a modified version of Langmuir model described for heterogenous system considering both low and high concentration of adsorbate (assuming most sites having lower energy)	[5]
Temkin	$q_e = B \ln A_T + B \ln C_e$	Considers the interaction between the adsorbent and the adsorbate by ignoring the extremely large and low concentration values- it assumes that adsorption heat of all molecules in the layer declines linearly rather than logarithmically	[6]
Dublin-Radushkevich	$Q_e = Q_m \exp\left(\frac{(RT \ln(1 + 1/C_e))^2}{-2E^2}\right)$	Associates the mechanism of adsorption to the distribution of Gaussian energy onto the heterogeneous surface	[7]

Table S3. *Studied kinetic models*

Kinetic models			
**Model name**	**Equation**	**Description**	**Reference**
Pseudo-first (PFO)	$\frac{dq_t}{dt} = k_1(q_e - q_t)$	Adsorption is the difference equilibrium adsorption and the adsorbed capacity at time multiplied by the rate constant of the adsorption. The rate of adsorption is proportional to this driving force linearly	[8]
Pseudo-second (PSO)	$q_t = \frac{q_e^2 k_2 t}{1+q_e k_2 t}$	Adsorption is the difference between the equilibrium adsorption capacity and the adsorbed capacity multiplied by the rate constant. However, in this model, the rate of adsorption is proportional to the square of the driving force indicating each adsorbate occupies two adsorption sites	[9]
Elovich	$q_t = \alpha \ln(a\alpha) + \alpha \ln t$	This model looks into this from a chemisorption kinetics perspective by describing the reduction in rate of adsorption due to increase in surface coverage with time	[10]
Avrami's exponential	$q_t = q_e(1 - e^{-k_1 t})^n$	This model is adapted from Avrami's kinetic decomposition model which is used to evaluate the reaction rate as the fraction of adsorption at time, and the rate constant. It also considers multiple adsorption sites	[11]
Weber and Morris Diffusion	$q_t = k_p t^{\frac{1}{2}} + C$	The equation for the Weber and Morris intraparticle diffusion model is based on some assumptions. Firstly, it assumes that the resistance to mass transfer is only significant at the beginning of the diffusion. Secondly, the concentration governs the radial diffusion process, only constant diffusion occurs in the process	[12]
Diffusion Chemisorption (DC)	$\frac{t^n}{q_t} = \frac{1}{k_{DC}} + \frac{1}{q_e} t^n$	The diffusion-chemisorption model can be used to describe the sorption of adsorbate onto the heterogeneous surface. The model correlates the rate of change of concentration in solid phase to the rate of mass of transfer of pollutant in fluid phase to the biosorption side	[13]
Bangham	$\log\left[\log\left(\frac{C_0}{C_0 - q_t m}\right)\right] = \log\left(\frac{K_j m}{2.303 V}\right) + \alpha \log t$	It is a logarithmic model used to evaluate the ability of pore diffusion in the adsorption process	[14]

Table S4. *Thermodynamic calculation for MB adsorption by KBS*

Temp: 313.5K				Temp: 303.15 K				Temp: 298.15 K			
Ce (ppm)	qe (mg/g)	K_d	ln (K_d)	Ce (ppm)	qe (mg/g)	Kd	ln (Kd)	Ce (ppm)	qe (mg/g)	Kd	ln (Kd)
8.23	11.77	1.430	0.357	10.13	9.87	0.974	-0.026	12.43	7.57	0.609	-0.495
18.19	21.81	1.199	0.181	21.19	18.81	0.887	-0.119	29.19	10.81	0.370	-0.993
30.36	29.64	0.976	-0.024	35.36	24.64	0.696	-0.361	44.36	15.64	0.352	-1.042
46.34	33.66	0.726	-0.319	49.34	30.66	0.621	-0.475	59.34	20.66	0.348	-1.055
62.12	37.88	0.609	-0.494	66.12	33.88	0.512	-0.668	74.52	25.48	0.341	-1.073
105.02	44.98	0.428	-0.845	111.02	38.98	0.351	-1.046	120.02	29.98	0.249	-1.387
150.18	49.82	0.331	-1.103	159.18	40.82	0.256	-1.360	168.18	31.82	0.189	-1.664
196.03	53.97	0.275	-1.289	207.03	42.97	0.207	-1.572	217.03	32.97	0.152	-1.884
242.673	57.327	0.236	-1.443	254.673	45.327	0.178	-1.726	266.673	33.327	0.125	-2.079
341.212	58.788	0.172	-1.758	353.56	46.44	0.131	-2.029	365.56	34.44	0.094	-2.362
440.73	59.27	0.134	-2.006*	453.46	46.54	0.102	-2.276*	465.46	34.54	0.074	-2.600*

**K_d values used for the Arrhenius plot for thermodynamic calculations*

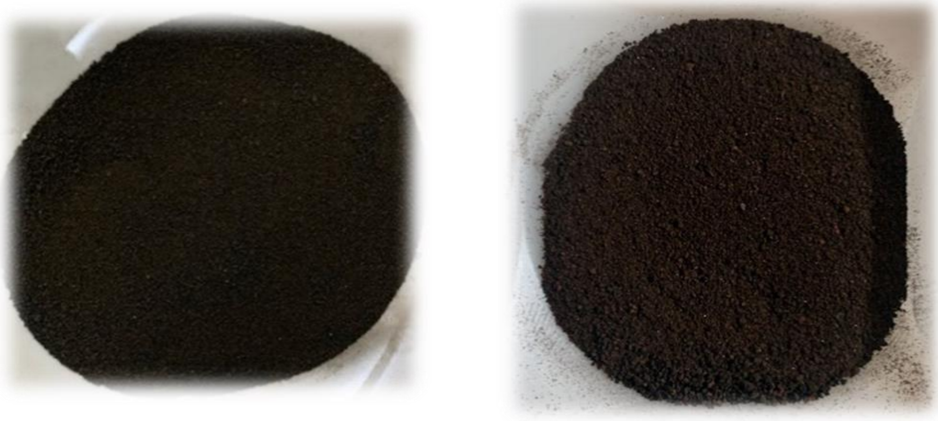

(a) (b)

Figure S1. Biosolids (a) before (BS) and (b) after activation (KBS)

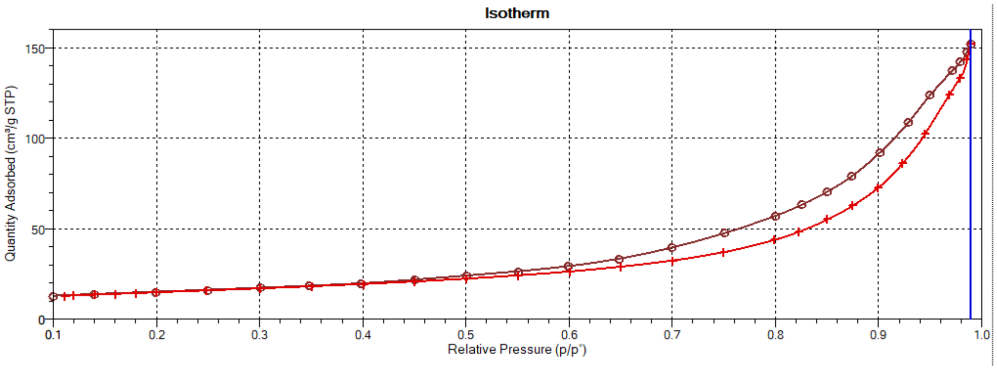

Figure S2. N_2 adsorption of KBS using BET instrument

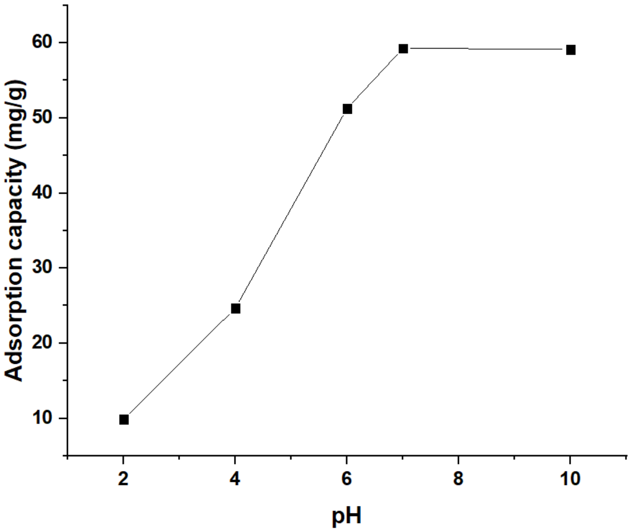

Figure S3. Effect of pH on methylene blue adosprtion

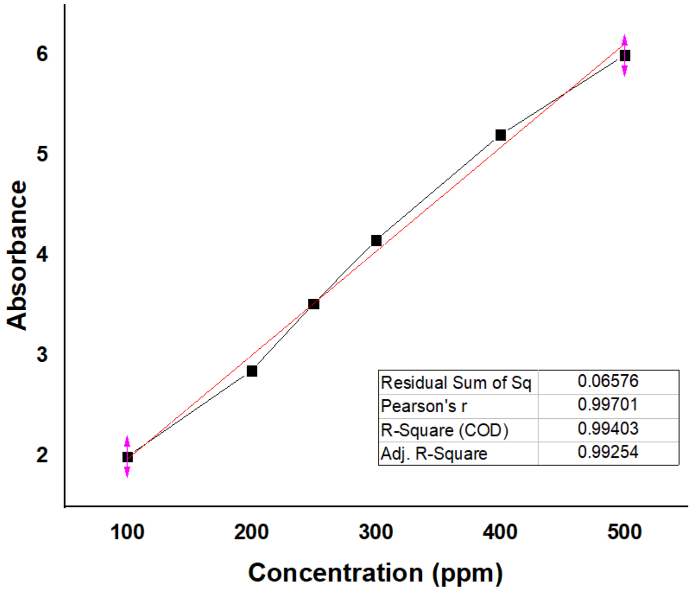

Figure S4. Methylene blue calibration curve using UV-Vis Spectophotometer

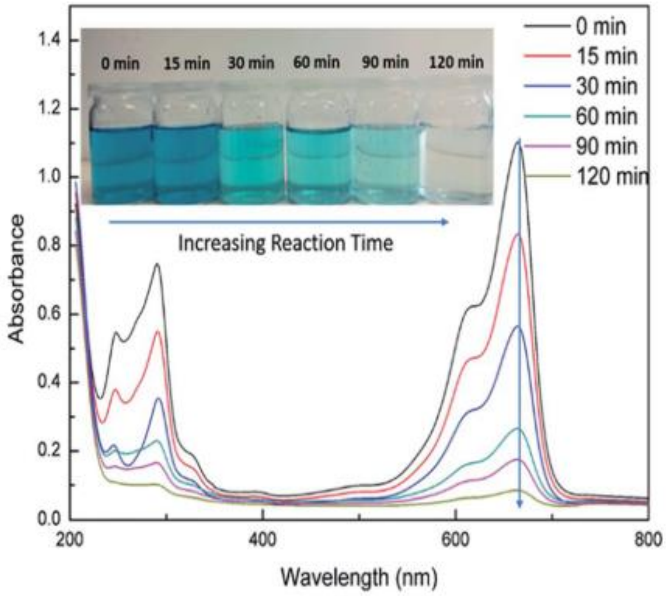

Figure S5. Methylene blue UV-Vis adsorption spectra extracted from [15]*

* *This article is an open access article distributed under the terms and conditions of the Creative Commons Attribution (CC BY) license*

References

1. Langmuir, I. A New Adsorption Isotherm. *Langmuir, I.* **1918**, *40*, 1361–1403.
2. Freundlich, H. Colloid and Capillary Chemistry. *London:Methuen* **1926**, 6–8.
3. Redlich, O. J. D. L., & Peterson, D.L. A Useful Adsorption Isotherm. *J. Phys. Chem.* **1959**, *63*, 1024–1024.
4. Sips, R. On the Structure of a Catalyst Surface. *J. Chem. Phys.* **1948**, *16*, 490–495.
5. Toth, J. Groundwater discharge: A common generator of diverse geologic and morphologic phenomena. *Int. Assoc. Sci. Hydrol. Bull.* **1971**, *16*, 7–24, doi:10.1080/02626667109493029.
6. Temkin, M.I. Kinetics of Heterogeneous Catalysis. *J. Phys. Chem.* **1940**, *14*, 1153–1158.
7. Dubinin, M.M. Surface and Nanomolecular Catalysis. *Zhur Phys Chem* **1960**, *34*, 959.
8. Lagergren, S. Zur Theorie Der Sogenannten Adsorption Gelo¨ster Stoffe. *K. Sven. Vetenskapsakademiens* **1898**, *24*, 1–39.
9. Ho, Y. S., & McKay, G. Pseudo-Second Order Model for Sorption Processes. *Process Biochem.* **1999**, *34*, 451–465.
10. Chien, S. H., & Clayton, W.R.(Application of Elovich Equation to the Kinetics of Phosphate Release and Sorption in Soils. *Soil Sci. Soc. Am. J.* **1980**, *44*, 265–268.
11. Lopes, E. C., dos Anjos, F. S., Vieira, E. F., & Cestari, A.R. An Alternative Avrami Equation to Evaluate Kinetic Parameters of the Interaction of Hg (II) with Thin Chitosan Membranes. *J. Colloid Interface Sci.* **2003**, *263*, 542–547.
12. Weber Jr, W. J., & Morris, J.C.(Kinetics of Adsorption on Carbon from Solution. J. *J. Div. Sanit. Eng.* **1963**, *89*, 31–59.
13. Hameed, B.H.; El-Khaiary, M.I. Equilibrium, Kinetics and Mechanism of Malachite Green Adsorption on Activated Carbon Prepared from Bamboo by K2CO3 Activation and Subsequent Gasification with CO2. *J. Hazard. Mater.* **2008**, *157*, 344–351, doi:10.1016/j.jhazmat.2007.12.105.
14. Aharoni, C., Ungarish, M. Kinetics of Activated Chemisorption Part 2. — Theoretical Models. *J. Chem. Soc. Faraday Trans* **1977**, *73*, 456–464.
15. Khan, I.; Saeed, K.; Zekker, I.; Zhang, B.; Hendi, A.H.; Ahmad, A.; Ahmad, S.; Zada, N.; Ahmad, H.; Shah, L.A.; et al. Review on Methylene Blue: Its Properties, Uses, Toxicity and Photodegradation. *Water (Switzerland)* **2022**, *14*, doi:10.3390/w14020242.
